# Data on the effect of homogenization heat treatments on the cast structure and tensile properties of alloy 718Plus in the presence of grain-boundary elements

**DOI:** 10.1016/j.dib.2017.06.055

**Published:** 2017-07-04

**Authors:** Seyed Ali Hosseini, Karim Zangeneh Madar, Seyed Mehdi Abbasi

**Affiliations:** Metallic Materials Research Center, Malek Ashtar University of Technology (MUT), Tehran, Iran

**Keywords:** Homogenization, Boron, Zirconium, 718Plus, Strength, Ductility

## Abstract

The segregation of the elements during solidification and the direct formation of destructive phases such as Laves from the liquid, result in in-homogeneity of the cast structure and degradation of mechanical properties. Homogenization heat treatment is one of the ways to eliminate destructive Laves from the cast structure of superalloys such as 718Plus. The collected data presents the effect of homogenization treatment conditions on the cast structure, hardness, and tensile properties of the alloy 718Plus in the presence of boron and zirconium additives. For this purpose, five alloys with different contents of boron and zirconium were cast by VIM/VAR process and then were homogenized at various conditions. The microstructural investigation by OM and SEM and phase analysis by XRD were done and then hardness and tensile tests were performed on the homogenized alloys.

**Specifications Table**

**Table:** 

Subject area	Materials Science and Engineering
More specific subject area	Cast structure characterization and tensile properties of alloy 718Plus
Type of data	Figure and Table
How data was acquired	The microstructural investigation by LEICA MEF4A Optical Microscopy and ZEISS SUPRA^TM^ 55 Scanning Electron Microscopy and phase analysis by XRD (Inel Equinox 6000) were done and then hardness and high-temperature tensile tests were performed on the alloys (MVK-HO and ATM CR-100KNB machine tests were used respectively).
Data format	Analyzed
Experimental factors	The double vacuum melting process VIM/VAR was used to produce the alloy 718Plus ingots with different amounts of boron (0.00–0.016 wt%) and zirconium (0.0–0.1 wt%). The homogenization treatment of alloys was carried out in the temperature range 1075–1175 °C for 5–25 h based on the DoE method.
Experimental features	The alloy structure was examined by OM and SEM and X-Ray diffraction analysis was used in order to more accurately evaluate the type and composition of the phases. The hardness test was carried out according to ASTM E384-99 (micro-hardness) [1] and ASTM E18-03 (macro-hardness) [2]. The hot tensile test was performed on homogenized alloys at two temperatures of 704 °C and 1100 °C and two strain rates of 0.02 and 0.06 min^−1^ respectively, according to ASTM E21 [3].
Data source location	Malek Ashtar University of Technology, Tehran, Iran.
Data accessibility	Data is with this article

**Value of the data**

• These collected data can be useful for other researchers in order to select the optimal homogenization heat treatment conditions of alloy 718Plus and the similar alloys like In718.
• The data about the cast structure and tensile properties variation in the presence of boron and zirconium can be useful for using of these elements in other superalloys.
• The data can be useful for the selection of the condition of homogenization treatment in the present of boron and zirconium, in order to maintain the mechanical properties of superalloys.

## 1 Data

The SEM images of the cast structure of alloy 718Plus with different amounts of boron and zirconium and the results of the XRD analysis are presented here. In addition, the results of the hot tensile test that was performed on the cast alloy 718Plus in different homogenization conditions were reported. The effect of boron and zirconium on the tensile properties of the alloy 718Plus under the same homogenization condition are the other part of the data.

## 2 Experimental design, materials and methods

**Fig. 1:**
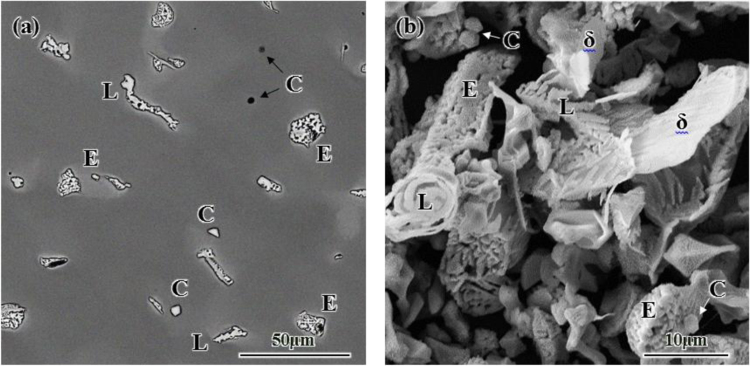
SEM images of a) the cast structure of the base alloy 718Plus, b) the phases extracted from matrix (L: Laves, E: eutectic Laves-gamma, C: niobium carbide, D: titanium carbide and *δ*: delta).

**Fig. 2:**
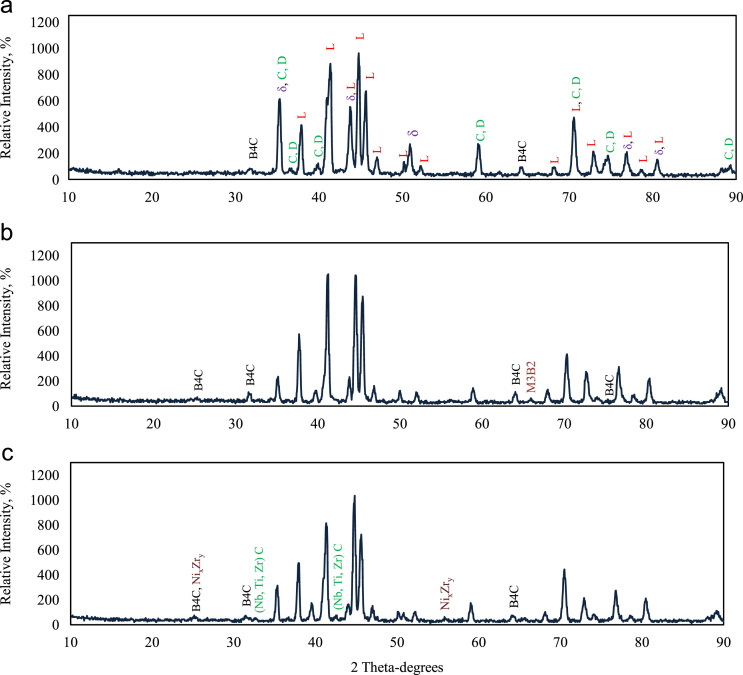
The XRD results obtained from the phases extracted from the alloys a) 718Plus (4B), b) containing boron (16B), c) containing zirconium (4B100Z) (L: Laves, C: niobium carbide, D: titanium carbide, and *δ*: delta and gamma prime).

The alloys structure was examined by Optical Microscopy and Scanning Electron Microscopy, operated at 15 kV. The SEM results of the cast structure of alloy 718Plus with its extracted phases are shown in Fig. 1. These phases include titanium and niobium carbides, Laves, eutectic Laves-gamma and small amounts of the delta. Fig. 2 shows the results of the XRD analysis of the extracted phases from 16B (0.016 wt% boron) and 4B100Z (0.1 wt% zirconium) with compared to the base alloy.

**Fig. 3:**
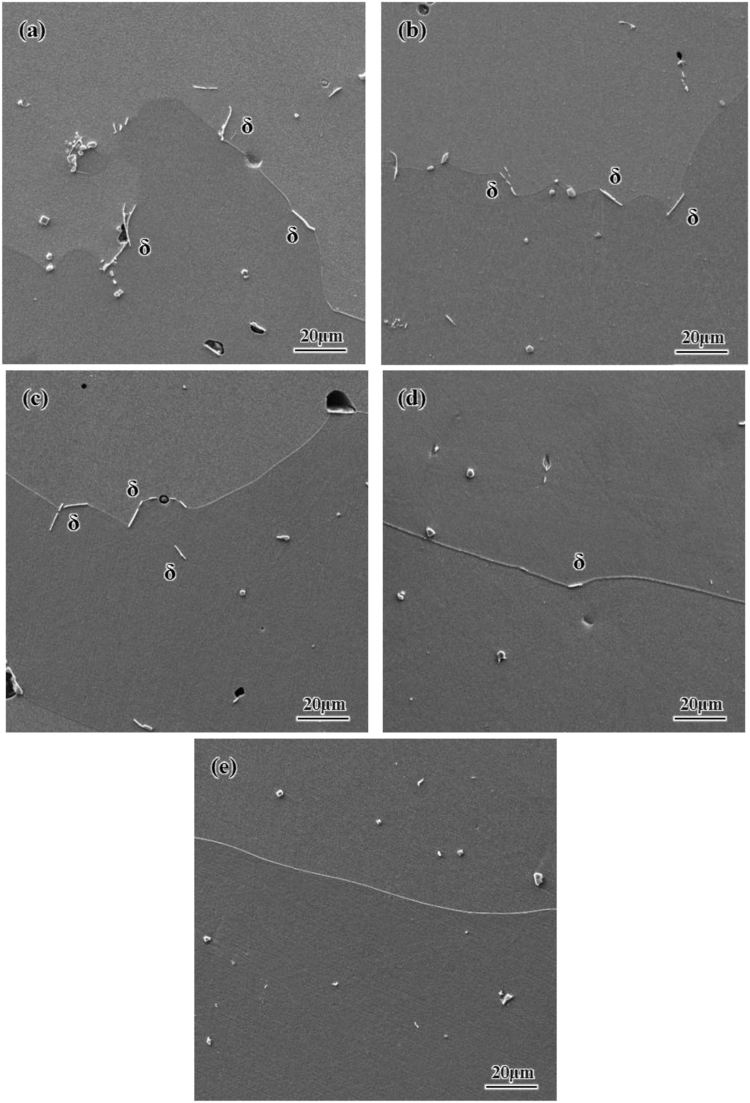
The amount of delta phase which decreased on the grain boundaries as increasing the time and temperature of homogenization for the alloys; a) H11, b) H12, c) H13, d) H14, e) H15.

Fig. 3 shows the variation of intergranular plate-like delta phase at high time and temperature of homogenization.

**Fig. 4:**
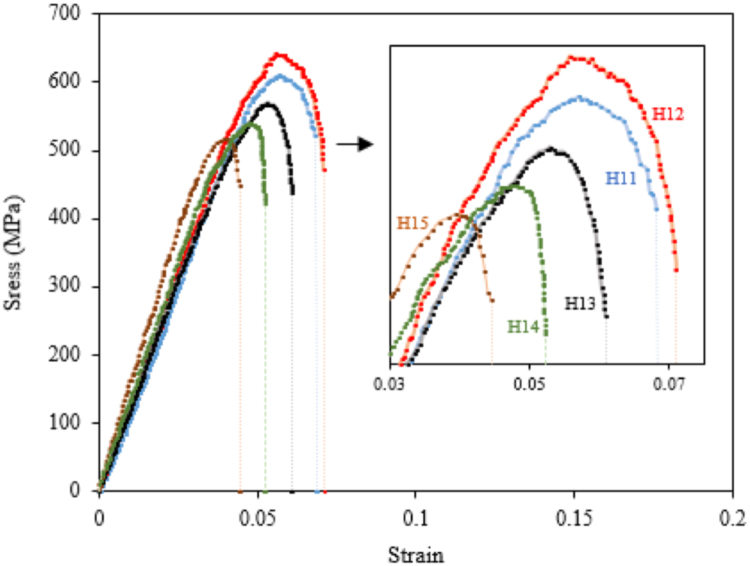
The hot tensile test results at 704 °C and 0.02 min^−1^ strain rate for different homogenization conditions.

The results of the macro-hardness test are presented in Table 1. The hot tensile tests were performed at 704 °C as the service temperature of the cast alloy 718Plus in different homogenization treatment conditions. The results of this test are presented in Fig. 4.

**Table 1:** The results of macro-hardness test of the alloys before homogenization treatment (Vickers).

**Alloy code**	**4B**	**16B**	**4B100Z**	**16B100Z**	**10B50Z**
**Hardness**	350±3	375±3	374±3	385±3	364±3

**Fig. 5:**
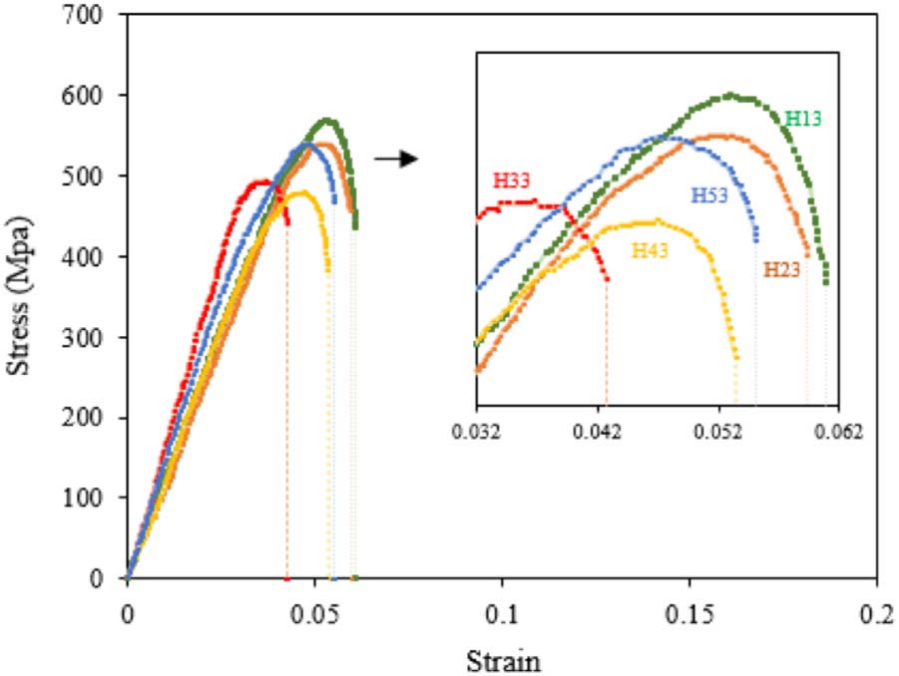
The hot tensile test results at 704 °C with a 0.02 min^−^^1^ strain rate for the alloys containing Boron and Zirconium in 1125 °C-10 h homogenization condition.

The effect of boron and zirconium on the hot tensile properties of the alloy 718Plus under the same 1125 °C-10 h homogenization conditions is presented in Fig. 5.
